# Identifying social media professionalism competencies in Vietnamese medical students: a modified Delphi method

**DOI:** 10.3389/fpubh.2026.1703842

**Published:** 2026-02-11

**Authors:** Thi Kim Chi Dang, Byung-Il Yeh, Yon Chul Park, Bui Bao Hoang, Khanh Vinh, Kyung Hye Park

**Affiliations:** 1 Skills Lab Center, Undergraduate Training Office, University of Medicine and Pharmacy, Hue University, Hue, Vietnam; 2 Department of Medical Education, Yonsei University Wonju College of Medicine, Wonju, Republic of Korea; 3 Department of Internal Medicine, University of Medicine and Pharmacy, Hue University, Hue, Vietnam; 4 Undergraduate Training Office, Department of Internal Medicine, University of Medicine and Pharmacy, Hue University, Hue, Vietnam

**Keywords:** Delphi method, medical education, medical students, professionalism, social media

## Abstract

**Introduction:**

Medical education in Vietnam is advancing toward global standards, emphasizing professionalism from the earliest stages of training. However, the growing use of social media presents new challenges to professionalism, as inappropriate and unprofessional online behavior can negatively impact students’ future careers, breach patient confidentiality, and erode public trust. Despite these concerns, no official guidelines or educational opportunities regarding professionalism on social media (PSM) are currently available for medical students in Vietnam, including at Hue University of Medicine and Pharmacy (Hue UMP). This study aimed to reach consensus among experts on the competencies in PSM that Vietnamese medical students should acquire.

**Methods:**

A modified Delphi survey with two rounds was conducted from April to May 2025. A literature review was performed to identify the initial competency list. Thirty-two Hue UMP participants rated items on a 5-point Likert scale via Google Forms. Consensus was determined using Lawshe’s content validity ratio (CVR).

**Results:**

Thirty-nine competencies in five domains were identified. After two rounds of Delphi surveys using CVR thresholds for inclusion, experts reached consensus on 23 key competencies for PSM for medical students in Vietnam. These included competencies spanned five domains: patient confidentiality, privacy, and dignity; professional boundaries, doctor-patient relationship, and public trust; practitioner’s privacy; health advocacy; and information appropriateness. These results can be used to develop curriculum or guidelines for the professional use of SM for medical students and physicians.

**Discussion:**

Experts reached consensus on 23 PSM competencies across five domains as essential for Vietnamese medical students. These competencies provide a foundation for developing educational interventions in PSM to support responsible online conduct, protect patient rights, and maintain public trust in the medical professions.

## 1 Introduction

Social media (SM) has revolutionized health services (1), transforming how healthcare professionals and students communicate and collaborate. It serves as a reference for sensitive health issues contributed by both nonprofessionals and physicians (2). As a digital medium, SM fosters online communities where users exchange ideas and content (3). SM can be grouped into five types: collaborative projects (e.g., Wikipedia), blogs and microblogs (e.g., Blogger, Twitter), content-sharing communities (e.g., YouTube), social networking sites (e.g., Facebook), and virtual worlds (e.g., HumanSim). These platforms often display lists of connected users, allowing visibility into activities and connections (1, 4).

The use of SM by medical doctors and medical students is increasing both personally and professionally (5). According to 2025 statistics, 91% of healthcare professionals worldwide use SM for professional purposes, of which 65% use it for patient education (6). Additionally, studies have shown that 90% of medical doctors use some form of SM; of these, 60% of them stated that SM has positively influenced the quality of patient care they provide (6, 7). In the medical field, health professionals utilize SM for various purposes (8–11). These include exchanging information about professional problems and clinical experiences, networking, education, marketing, promotion, patient care, and awareness campaigns (8–11). Moreover, SM is a significant part of medical students’ lives, as they belong to the “net generation” and have grown up with these platforms for various beneficial purposes, including sharing experiences and building supportive peer network (12). Surveys conducted at medical schools in Vietnam showed that over 99% of medical students use SM frequently, with 90% reporting daily use for learning purposes (13, 14).

While the use of SM presents many benefits for medical doctors and patients, it has also raised concerns about professionalism, including breaches of patient privacy and confidentiality, blurred professional boundaries between patient and physician, damage to professional image and public trust, and dissemination of poor-quality information (2, 15, 16). Studies have found that many health professionals and medical students engage with others on SM in an unprofessional and inappropriate way and have encountered problems with their online identities (17, 18). Further, multiple studies have revealed instances of vulgar or derogatory language, breaches of patient confidentiality, and sharing of inappropriate content, which can even lead to legal issues for medical students or medical doctors when using SM (19).

To address these challenges, many healthcare authorities and regulatory bodies worldwide have issued professional standards, guidelines, evidence-based reports, and consensus statements on the professional use of SM (20). Although the term PSM is not explicitly used in these documents, the concept is implicitly addressed when professional behavior is discussed in the SM context (21). While there is no single, universally accepted definition of PSM, common principles have been consistently emphasized across professional guidelines and academic literature (22). PSM generally refers to maintaining ethical, respectful, and responsible behavior in online environments, aligning with the standards expected in professional practice and traditional medical professionalism (23–25). The American Medical Association (AMA) defines PSM as maintaining appropriate boundaries, safeguarding patient privacy, and acting with honesty and respect in online interactions (26). Similarly, the General Medical Council (GMC) states that doctors’ online behavior must align with the professional standards required in face-to-face practice (27). As SM becomes increasingly integrated into healthcare communication and education, various professional organizations and studies have emphasized the importance of upholding PSM (22, 27–30). A framework of SM competencies for health professionals has previously been developed using a modified Delphi survey, in which professionalism within SM was identified as a key domain (31). This included components for posting or sharing content as well as responding to online threats by using privacy settings (31).

Moreover, PSM has been taught across medical, nursing, and allied health students (32). Some medical institutions have made efforts to provide medical students with PSM education through different approaches to curriculum development. One medical school, for example, has developed workshop and blog-based interventions to promote professionalism among medical students using SM (33). Another school has designed and implemented a 90 min educational session to raise students’ awareness of their new role as medical professionals and the implications for their SM presence (34). It is suggested that there is a link between being educated about professionalism and displaying more cautious behaviors online (35).

In Vietnam, across 32 medical schools, education on medical professionalism remains uncommon, with only five having recently introduced it into their curricula. These courses aim to provide medical students with the concepts and attributes that constitute medical professionalism, as well as the theoretical basis for developing professionalism in the medical education environment and in medical practice. However, current curricula do not address the specific dimensions of professionalism related to SM use, leaving a significant gap in the educational framework. Meanwhile, digital competency has become essential following the Vietnamese Ministry of Education and Training’s publication of the Regulations on the Digital Competency Framework for Learners in January 2025 (36). This document establishes a foundation for developing training standards and educational programs on digital competencies for learners. Consequently, medical schools are now preparing to develop new modules to address these emerging requirements. Furthermore, the rapid growth of SM use among medical students has raised concerns regarding professional behavior online. Despite this, there is a notable absence of guidance and policies addressing the appropriate use of SM, as well as of a PSM framework to direct education on the professional use of SM for medical students. Therefore, this study aims to define, through expert consensus, the PSM competencies that medical students in Vietnam should acquire.

## 2 Materials and methods

### 2.1 Research design

This cross-sectional descriptive study utilized a modified two-round Delphi method to develop a competency list for PSM among Vietnamese medical students.

**Figure 1:**
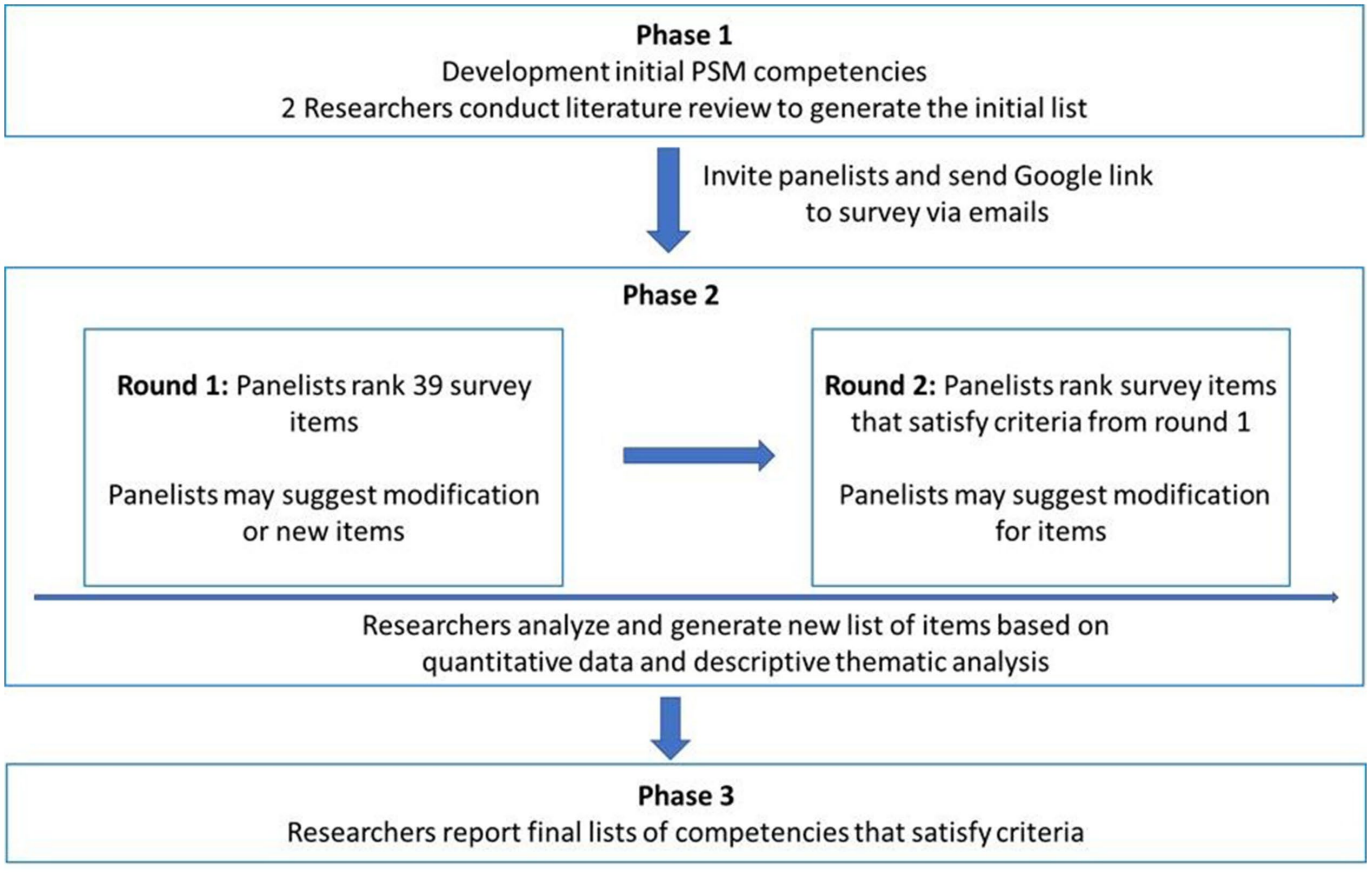
Overall research design.

A multiphase, modified Delphi survey was conducted from April to May 2025 to identify competencies in PSM for medical students and reach consensus on the most important competencies. Each panelist completed the survey online via Google Forms. Panelists conducted survey questionnaires via anonymized emails to avoid bias. The following research procedures were conducted to reach a consensus on PSM competencies (Figure 1). First, a literature review was performed to construct the initial draft of the items for PSM competencies. While the classic Delphi method uses an unstructured questionnaire to explore and converge on survey items before commencing the actual survey, the modified Delphi method establishes a list of items by reviewing relevant literature beforehand, bypassing this exploratory phase. Second, a two-round survey was performed wherein panelists evaluated the validity of PSM competencies. Delphi studies are commonly done in two to three rounds (37, 38) and this study was conducted in two rounds instead of three because the panel is well-informed and the initial item set is clearly defined. Only those who completed the round 1 Delphi survey were invited to participate in round 2. Finally, the researchers concluded the survey and achieved consensus.

### 2.2 Research subjects

Purposive sampling method was used to select the panelists for this study. The consensus was sought from panelists engaged in teaching and training medical students at Hue UMP and its teaching hospital, including full-time lecturers, lecturers-doctors, administrators, medical education specialists, nurses, and residents. Medical students and patients were excluded because the study focused on expert consensus among those responsible for curriculum design, assessment, and professional standards. To gain a comprehensive perspective, candidates with varied expertise and training experience were invited from the Department of Internal Medicine, Surgery, Obstetrics and Gynecology, Pediatrics, Oncology, Psychiatry, and Emergency Medicine; Faculty of Family Medicine; Faculty of Public Health; Undergraduate Training Office; and Student Affairs Office. Although the Delphi method does not state a predetermined number of panelists, the recommended number to achieve consensus in healthcare is 20 (39). Conclusion criteria include candidates engaging in teaching and training medical students at Hue UMP and the teaching hospital of Hue UMP, fully understanding the purpose and method of this study and the survey, and giving informed consent and volunteering to participate in this study. Emails were sent to 32 candidates who met these criteria to explain the study and to confirm their agreement to participate as panelists. Candidates who refused to give informed consent were excluded from the study. Responses deemed to have an inaccurate effect on the research results owing to insincerity were excluded.

This study was approved by the Institutional Review Board (IRB) of Yonsei University, Wonju Severance Christian Hospital (IRB approval no. CR325003), and the Ethics Committee in Biomedical Research of Hue UMP (approval no. H2025/053). Since the survey was conducted via an online service, the requirement to sign a written consent form was omitted. Instead, an explanation was provided in an online survey detailing the purpose and process of the study, roles of panelists, and personal information collected. Participants indicated their consent by clicking the consent checkbox on the online survey form for the collection of personal information. Personal information was collected to the minimum extent necessary for data processing, provided it could not be used to identify the individual.

### 2.3 Survey items development

The survey comprised two domains: (1) socio-demographic data and (2) competency in PSM. Information was collected about the participant’s gender, current professional role, working experience in the current role, and current department in which the respondent works.

This study was initiated by generating a list of PSM competencies for medical students from the existing literature, global guidelines on SM uses for medical students and medical doctors, and Vietnamese regulations on the digital competency framework for learners (40). Guidelines from both English-speaking and non-English-speaking countries were reviewed, including those issued by the AMA Council on Ethical and Judicial Affairs (41), Australia Medical Association (40), Canada Federation of Medical Students (42), GMC (43), Health Professions Council of South Africa (44), Korean Medical Association (45), Hong Kong Academy of Medicine Professionalism and Ethics Committee (45), Indian National Medical Council (46), Sri Lanka Medical Council (47), and World Medical Association (48). Thematic analysis was conducted on these documents to identify key principles and themes. A list of 39 competency items was extracted from the literature review and categorized into 5 domains: Patient confidentiality, privacy, and dignity; Professional boundaries, doctor-patient relationship, and public trust; Practitioner’s privacy; Health advocacy; and Information appropriateness. Each item consisted of a description of the competency, followed by a 5-point Likert scale (1 = Strongly disagree, 2 = Disagree, 3 = Neutral, 4 = Agree, 5 = Strongly agree) for participants to indicate their extent of agreement with the need for medical students to have that competency. In addition, panelists were given the opportunity to use their discretion to include additional competencies or suggestions for modification after each competency domain.

### 2.4 Data collection

In the first round, a web-based questionnaire was emailed to the panelists. They were asked to evaluate the validity of 39 PSM competencies on a 5-point scale, identify redundancies, propose additional items, and suggest changes in wording or the integration of competencies. After collecting the data, qualitative analysis of the free-text suggestions was performed during post-meeting by two researchers (Thi Kim Chi Dang, Kyung Hye Park). During the post-meeting, based on the panelists’ opinions, revisions or exclusions of competency items that did not meet the inclusion criteria were discussed. Items that met the inclusion criteria for the first round were thematically analyzed and incorporated as new items in the second round.

As with the first round, the emails were sent to panelists with an attached link to the online survey. The panelists were asked to evaluate the validity (5-point scale) of a new list of items derived from the results of the first round and to write their opinions on potential modifications. In addition to the list of items, a detailed thematic analysis and summary of results from the first round were provided in a PDF, allowing the panelists to review details of the items and their corresponding codes. A post-meeting was conducted by two researchers (Thi Kim Chi Dang, Kyung Hye Park) after the second round to discuss and finalize the list of competencies based on the inclusion criteria and thematic analysis, as in the first round.

### 2.5 Data analysis

The collected data were exported and stored in Microsoft Excel. 16.0 (Microsoft Corp, Redmond, United States) for initial organization, cleaning, and statistical analysis. To determine the adequacy of the panelist’s evaluation of validity for the competencies in each domain, the mean, standard deviation, degree of convergence, degree of consensus, and CVR were analyzed.

The formula for calculating the degree of convergence is as follows:


$$\frac{Q3-Q1}{2}$$

where Q3 = the 3rd quartile coefficient, Q1 = the 1st quartile coefficient.

The degree of convergence is an index that shows whether the response results gathered from the Delphi survey are converging (43). The convergence degree increases when it is closer to 0; when it is between 0 and 0.5, the opinions of panelists are considered to be converging.

The formula for calculating the degree of consensus is as follows:


$$\frac{1-(Q3-Q1)}{\text{Median}}$$

The degree of consensus increases when it is closer to 1; when it is 0.75 or higher, the opinions of panelists are considered to reach a consensus.

The content validity ratio was calculated as follows:


$$\mathrm{CVR}=\frac{\left(\mathrm{ne}-(N/2)\right)}{N/2}$$

where ne = the number of panel members indicating that an item is essential.

In this study, ne is the number of respondents who rate an item as “Agree (4 points)” and “Strongly agree (5 points)” on a 5-point Likert scale, *N* = the number of panel members, CVR is evaluation of whether the items included in a survey truly reflect the content that is relevant to the concept being measured (49). Furthermore, descriptive analysis of panelists’ opinions, such as integration or exclusion of each competency, was conducted to reflect the results. Based on Lawshe’s critical values, the minimum acceptable CVR threshold was determined according to panel size, with a cutoff value of 0.37 for the first Delphi round (*N* = 29) and 0.42 for the second round (*N* = 24) (49).

## 3 Results

### 3.1 Results of the first-round Delphi survey

#### 3.1.1 Panelist demographics

The first-round survey was sent to 32 people on April 22nd. Twenty-nine participants completed it (91% response rate) between April 22nd and April 29th. The panel was nearly gender-balanced, with 15 (51.7%) female and 14 (48.3%) male participants. The most common professional role was doctor-lecturer (*n* = 17, 58.6%), followed by administrator (*n* = 4, 13.8%), lecturer (*n* = 3, 10.3%), resident (*n* = 3, 10.3%), and nurse (*n* = 2, 6.9%). Participants were affiliated with a variety of departments, faculty, and offices, with most belonging to the Departments of Pediatrics (5; 17.2%), followed by the Departments of Obstetrics and Gynecology and Internal Medicine (4 each; 13.8% each). Regarding professional working experience, 24 participants (82.8%) had more than 5 years of experience in medical education (Table 1).

**Table 1:** Demographic characteristics of the panelists in the first-round.

Characteristics	Frequency (%)	
	Round 1	Round 2
Gender		
Male	14 (48.3)	10 (41.7)
Female	15 (51.7)	14 (58.3)
Current professional role		
Lecturer	3 (10.3)	2 (8.3)
Doctor-Lecturer	17 (58.6)	16 (66.7)
Administrator	4 (13.8)	3 (12.5)
Resident	3 (10.3)	2 (8.3)
Nurse	2 (6.9)	1 (4.2)
Working experience		
<2 years	1 (3.4)	1 (4.2)
2–5 years	4 (13.8)	4 (16.7)
5–10 years	12 (41.4)	11 (45.8)
>10 years	12 (41.4)	8 (33.3)
Affiliation		
Department of internal medicine	4 (13.8)	3 (12.5)
Department of surgery	2 (6.9)	2 (8.3)
Department of obstetrics and gynecology	4 (13.8)	3 (12.5)
Department of pediatrics	5 (17.2)	4 (16.7)
Department of oncology	1 (3.4)	1 (4.2)
Department of psychiatry	1 (3.4)	1 (4.2)
Department of emergency	2 (6.9)	2 (8.3)
Faculty of public health	2 (6.9)	2 (8.3)
Faculty of family medicine	3 (10.3)	3 (12.5)
Undergraduate training office	2 (6.9)	2 (8.3)
Student affairs office	1 (3.4)	1 (4.2)
No respond	2 (6.9)	0 (0)

#### 3.1.2 Competencies

The results of the first-round Delphi survey were analyzed according to the following inclusion criteria: (1) CVR threshold is 0.37 or higher, (2) degree of convergence is 0–0.5, and (3) degree of consensus is 0.75 or higher. Descriptive opinions, such as integration, rewording, or exclusion of each competency, were also incorporated (Table 2). After round 1, 23 items met the inclusion criteria. Among these, 15 remained as they were, while 17 with similar meanings were combined into 8; 7 were removed from the list because they did not meet the inclusion criteria. Finally, 39 competencies across 5 domains were restructured into 23 competencies across 5 domains.

**Table 2:** Content validation index for PSM competency and expert’s consensus in the first round.

Domain	Competency	Mean ± SD	Degree of convergence	Degree of consensus	CVR^a^	Experts’ consensus
I. Patient confidentiality, privacy and dignity	1. I understand that ethical guidelines and laws related to patient privacy and confidentiality must be maintained online	4.62 ± 0.49	0.5	0.8	1.00	Included (integrated with competency 2)
	2. I maintain patient confidentiality and privacy online	4.66 ± 0.48	0.5	0.8	1.00	Included
	3. I refrain from sharing identifiable patient information on SM without explicit consent.	4.38 ± 0.68	0.5	0.75	0.93	Included (integrated with competency 5)
	4. I understand that even non-identifiable information, when combined, can lead to breaches of confidentiality.	3.45 ± 1.18	0.5	0.75	0.24	Excluded
	5. I obtain explicit written consent before sharing patient information online	4.17 ± 0.66	0.5	0.75	0.72	Included
	6. I disclose patient information only when it aligns with a court order, patient consent, and the law	4.69 ± 0.47	0.5	0.8	1.00	Included (integrated with competency 10)
	7. I use SM responsibly for academic exchange or education, ensuring patient information is anonymized and shared ethically	4.52 ± 0.63	0.5	0.8	0.86	Included
	8. I keep patient information confidential even after the patient dies.	4.14 ± 0.79	0.5	0.75	0.66	Included
	9. I am aware that there is always a risk that the information online can be disseminated even in “invisible groups”	4.00 ± 0.80	0.5	0.75	0.52	Included
	10. I limit the disclosure of patient information to the minimum necessary to keep patient’s privacy	4.41 ± 0.68	0.5	0.8	0.79	Included
II. Professional boundary, doctor-patient relationship, public trust	11. I maintain appropriate boundaries of the patient-physician relationship in accordance with professional ethics guidance	4.48 ± 0.57	0.5	0.8	0.93	Included
	12. I am aware of the risk where social and professional boundaries become unclear when using social media	4.17 ± 0.60	0.5	0.75	0.79	Included (integrated with competency 11)
	13. I distinguish between personal and professional content online	3.62 ± 1.32	1	0.5	0.31	Excluded
	14. I take steps to separate personal and professional content when necessary	3.90 ± 1.21	0.5	0.75	0.52	Included
	15. I communicate with patients on SM with mutual respect, professionalism, and trust	4.79 ± 0.41	0	1	1.00	Included
	16. I can communicate with colleagues on SM with mutual respect, professionalism, and trust	4.83 ± 0.38	0	1	1.00	Included
	17. I avoid abusive, discriminatory, or harmful behavior online	4.83 ± 0.38	0	1	1.00	Included
	18. I redirect patients seeking medical advice on SM to appropriate in-person care	3.83 ± 1.44	1	0.6	0.24	Excluded
	19. I handle inappropriate contact professionally	4.45 ± 0.74	0.5	0.8	0.72	Included
III. Practitioner’s privacy	20. I use privacy settings to safeguard personal information and content	4.34 ± 0.48	0.5	0.75	1.00	Included (integrated with competencies 21 and 22)
	21. I understand that privacy settings on SM are not absolute	4.14 ± 0.83	0.5	0.75	0.79	Included
	22. I understand that content I share online can be published without my intention	4.24 ± 0.64	0.5	0.75	0.79	Included
	23. I understand that content I share online can be permanent even I delete it from my account	3.55 ± 1.30	0.5	0.75	0.24	Excluded
	24. I separate personal and professional SM accounts to maintain appropriate boundaries	4.10 ± 0.86	0.5	0.75	0.72	Included
	25. I know how to limit the disclosure of my personal information	4.24 ± 0.87	0.5	0.75	0.79	Included (integrated with competency 26)
	26. I know how to control the extent of my SM posts	4.34 ± 0.94	0.5	0.8	0.72	Included
IV. Health advocacy	27. I understand that the use of SM may cause the conflict of interest	3.59 ± 1.32	1	0.5	0.24	Excluded
	28. I manage the sites based on understanding about conflict of interest caused by SM use	3.59 ± 1.27	1	0.5	0.17	Excluded
	29. I disclose any personal or financial interests when promoting or endorsing products, services, or healthcare practices on social media	4.45 ± 0.74	0.5	0.8	0.86	Included
	30. I avoid unfair promotion of medical services, practitioners, or products for financial or personal gain on social media	4.66 ± 0.48	0.5	0.8	1.00	Included
	31. I use SM responsibly for public health advocacy while maintaining professionalism and trust.	4.45 ± 0.69	0.5	0.8	0.79	Included
V. Information appropriateness	32. I ensure that all medical information I post on SM is appropriate, accurate and evidence-based	4.76 ± 0.51	0	1	0.93	Included (integrated with competency 33)
	33. I ensure that all medical information I post on SM is contextually appropriate to support public understanding and trust.	4.66 ± 0.55	0.5	0.8	0.93	Included
	34. I include sufficient context in my content to help the audience verify claims	4.62 ± 0.62	0.5	0.8	0.86	Included (integrated with competency 35)
	35. I include sufficient context in my content to help the audience understand the implications of the information shared	4.59 ± 0.57	0.5	0.8	0.93	Included
	36. I do not misrepresent my qualifications, experience, or expertise	4.72 ± 0.45	0.5	0.8	1.00	Included
	37. I actively monitor medical information on social media	3.66 ± 1.59	1	0.5	0.31	Excluded
	38. I endeavor to correct or supplement inaccurate or inappropriate content posted by myself	4.59 ± 0.73	0.5	0.8	0.86	Included
	39. I endeavor to correct or supplement inaccurate or inappropriate content posted by my colleagues	4.38 ± 0.82	0.5	0.8	0.59	Included

^a^CVR, Content validity ratio.

### 3.2 Results of the second-round Delphi survey

#### 3.2.1 Panelist demographics

The second-round survey was sent to 29 participants who fully participated in round 1 on April 30th. Of these, 24 participants completed round two of the Delphi survey (83% response rate) between April 30th and May 7th. The panel had a slight female majority, with 14 participants (58.3%) identifying as female and 10 (41.7%) as male. The predominant professional role was doctor-lecturer, accounting for 66.7% (*n* = 16) of participants, followed by administrator (*n* = 3, 12.5%), lecturer (*n* = 2, 8.3%), resident (*n* = 2, 8.3%), and nurse (*n* = 1, 4.2%). Participants were affiliated with various departments and offices, with the Department of Pediatrics being the most represented (*n* = 4, 16.7%), followed by the Department of Internal Medicine, Department of Obstetrics and Gynecology, and Faculty of Family Medicine (3 each, 12.5% each). Other affiliations included the Departments of Surgery and Emergency (*n* = 2 each, 8.3%); Faculty of Public Health (*n* = 2, 8.3%); and Departments of Oncology, Psychiatry, and the Student Affairs Office (*n* = 1 each, 4.2% each). A majority of the participants had professional experience of over 5 years, with 11 participants (45.8%) reporting 5–10 years and 8 participants (33.3%) reporting more than 10 years. Four participants (16.7%) had 2–5 years of experience, and only one participant (4.2%) had less than 2 years of experience (Table 1).

#### 3.2.2 Competencies

The results of the second-round Delphi survey were analyzed according to the following inclusion criteria: (1) CVR threshold is 0.42 or higher, (2) degree of convergence is 0–0.5, and (3) degree of consensus is 0.75 or higher. Panelists’ descriptive feedback—such as suggestions for integration, rewording, or exclusion of competencies—were thoroughly considered (Table 3). By the end of round two, all 23 items met the inclusion threshold. Of these, 22 items were remained unchanged, while one required modification. Specifically, the item “I do not misrepresent my qualifications, experience, or expertise” was revised to use active voice, as recommended by the panel. The revised version reads: “I accurately represent my qualifications, experience, and expertise.”

**Table 3:** Content validation index of the professionalism in SM competency and expert’s consensus in the second round.

Domain	Competency	Mean ± SD	Degree of convergence	Degree of consensus	CVR^a^	Experts’ consensus
I. Patient confidentiality, privacy and dignity	1. I maintain ethical guidelines and laws related to patient privacy and confidentiality online.	4.79 ± 0.41	0.00	1.00	1.00	Included
	2. I share identifiable patient information on SM only when I obtain explicit written consent from patient	4.79 ± 0.41	0.00	1.00	1.00	Included
	3. I disclose patient information to the minimum necessary like when it aligns with court order or when patient consent	4.83 ± 0.38	0.00	1.00	1.00	Included
	4. I use SM responsibly for academic exchange or education, ensuring patient information is anonymized and shared ethically	4.67 ± 0.48	0.50	0.80	1.00	Included
	5. I keep patient information confidential even after the patient dies.	4.71 ± 0.46	0.50	0.80	1.00	Included
	6. I am aware that there is always a risk that the information online can be disseminated even in “invisible groups”	4.83 ± 0.38	0.00	1.00	1.00	Included
II. Professional boundary, doctor-patient relationship, public trust	7. I maintain appropriate boundaries of the patient-physician relationship in accordance with professional ethics guidance	4.88 ± 0.34	0.00	1.00	1.00	Included
	8. I take steps to separate personal and professional content when necessary	4.75 ± 0.44	0.13	0.95	1.00	Included
	9. I communicate with patients on SM with mutual respect, professionalism, and trust	4.88 ± 0.34	0.00	1.00	1.00	Included
	10. I can communicate with colleagues on SM with mutual respect, professionalism, and trust	4.67 ± 0.48	0.50	0.80	1.00	Included
	11. I avoid abusive, discriminatory, or harmful behavior online	4.88 ± 0.45	0.00	1.00	0.92	Included
	12. I handle inappropriate contact professionally	4.88 ± 0.34	0.00	1.00	1.00	Included
III. Practitioner’s privacy	13. I use privacy settings to protect my personal information, while recognizing their limitations and the risk of unintended content disclosure	4.75 ± 0.44	0.13	0.95	1.00	Included
	14. I separate personal and professional SM accounts to maintain appropriate boundaries	4.83 ± 0.38	0.00	1.00	1.00	Included
	15. I know how to manage visibility and disclosure of my personal information and SM contents	4.79 ± 0.41	0.00	1.00	1.00	Included
IV. Health advocacy	16. I disclose any personal or financial interests when promoting or endorsing products, services, or healthcare practices on social media	4.75 ± 0.61	0.00	1.00	0.83	Included
	16. I avoid unfair promotion of medical services, practitioners, or products for financial or personal gain on social media	4.88 ± 0.45	0.00	1.00	0.92	Included
	17. I use SM responsibly for public health advocacy while maintaining professionalism and trust.	4.88 ± 0.45	0.00	1.00	0.92	Included
V. Information appropriateness	18. I ensure that all medical information I post on SM is accurate, evidence-based and contextually appropriate to support public understanding and trust.	4.88 ± 0.34	0.00	1.00	1.00	Included
	20. I include sufficient context in my content to help the audience verify claims and understand the implications	4.92 ± 0.28	0	1	1	Included
	21. I do not misrepresent my qualifications, experience, or expertise	4.92 ± 0.28	0	1	1	Modified and included
	22. I endeavor to correct or supplement inaccurate or inappropriate content posted by myself	4.96 ± 0.20	0	1	1	Included
	23. I endeavor to correct or supplement inaccurate or inappropriate content posted by my colleagues	4.79 ± 0.41	0	1	1	Included

^a^CVR, Content validity ratio.

Finally, after two rounds, 23 items in 5 domains were generated as competencies on PSM for medical students (Table 4).

**Table 4:** Final competency list on professionalism in social media.

Domain	Competency
I. Patient confidentiality, privacy and dignity	1. I maintain ethical guidelines and laws related to patient privacy and confidentiality online
	2. I share identifiable patient information on SM only when I obtain explicit written consent from patient
	3. I disclose patient information to the minimum necessary like when it aligns with court order or when patients consent
	4. I use SM responsibly for academic exchange or education, ensuring patient information is anonymized and shared ethically
	5. I keep patient information confidential even after the patient dies
	6. I am aware that there is always a risk that the information online can be disseminated even in “invisible groups”
II. Professional boundary, doctor-patient relationship, public trust	7. I maintain appropriate boundaries of the patient-physician relationship in accordance with professional ethics guidance
	8. I take steps to separate personal and professional content when necessary
	9. I communicate with patients on SM with mutual respect, professionalism, and trust
	10. I can communicate with colleagues on SM with mutual respect, professionalism, and trust
	11. I avoid abusive, discriminatory, or harmful behavior online
	12. I handle inappropriate contact professionally
III. Practitioner’s privacy	13. I use privacy settings to protect my personal information, while recognizing their limitations and the risk of unintended content disclosure
	14. I separate personal and professional SM accounts to maintain appropriate boundaries
	15. I know how to manage visibility and disclosure of my personal information and SM contents
IV. Health advocacy	16. I disclose any personal or financial interests when promoting or endorsing products, services, or healthcare practices on social media
	17. I avoid unfair promotion of medical services, practitioners, or products for financial or personal gain on social media
	18. I use SM responsibly for public health advocacy while maintaining professionalism and trust
V. Information appropriateness	19. I ensure that all medical information I post on SM is accurate, evidence-based and contextually appropriate to support public understanding and trust
	20. I include sufficient context in my content to help the audience verify claims and understand the implications
	21. I accurately represent my qualifications, experience, and expertise
	22. I endeavor to correct or supplement inaccurate or inappropriate content posted by myself
	23. I endeavor to correct or supplement inaccurate or inappropriate content posted by my colleagues

## 4 Discussion

Using a modified Delphi survey, the study identified competencies that medical students should acquire in PSM. Our findings align with prior international Delphi studies on SM competencies. While Yilmaz et al. developed a broad framework of 46 competencies across multiple domains, only six competencies addressed professionalism (31). By focusing exclusively on professionalism on SM, our study provides a more granular framework, resulting in 23 core competencies tailored to the ethical and professional challenges faced by medical students in digital contexts. Given the increasing use of SM among medical students and health professionals and the concerns about unprofessional behaviors online (15, 50–56), these findings provide an important foundation for developing training on PSM in medical schools. This is especially relevant in the context of medical education in Vietnam, where no official guidelines or defined competencies currently exist for the professional use of SM by medical students. This study was the first in Vietnam to identify PSM competencies to be included in the undergraduate medical curriculum, using a modified Delphi method among medical expert. The results of this study can be used to develop guidelines for the professional use of SM for medical students and physicians and to serve as a basis for developing a curriculum on PSM in Vietnamese medical schools. Comprehensive guidelines for SM use as well as educational opportunities for medical students are recommended to enhance professionalism on the online environment (21, 57).

The domain “Patient confidentiality, privacy and dignity” was most frequently mentioned in the literature review on PSM (41–44, 48, 58), indicating that it is important to have a basic domain of competencies in PSM. Accordingly, students must follow ethical and legal standards when handling patient information online. Identifiable information should only be shared with explicit consent and must always be anonymized for academic use. In addition, confidentiality extends beyond a patient’s death, and students should recognize that content shared online—even in private groups—can still spread unintentionally. This aligns with the core principles of medical professionalism education, which emphasizes the importance of patient confidentiality and respect for patients’ privacy and dignity (59, 60). This foundational value remains essential in the online environment, underscoring the need to extend traditional professionalism to contexts where the risks of breaching confidentiality are heightened. Notably, the item “I understand that even non-identifiable information, when combined, can lead to breaches of confidentiality” was excluded during the Delphi survey. This may reflect the Vietnamese context, wherein understanding of data privacy is still evolving and the distinction between identifiable and non-identifiable information is not always clearly emphasized. Panelists may assume that removing a patient’s name or direct identifiers is sufficient, without recognizing that combinations of data, such as age, condition, location, and time, can still lead to re-identification, especially in local or community settings. Although the disclosure of such information is prohibited under Vietnamese laws on medical confidentiality (61), it may not be widely recognized or understood by health professionals in Vietnam.

In the “Professional boundary, doctor-patient relationship, and public trust” domain, maintaining clear boundaries and separating personal from professional content is essential. Communication with patients and colleagues must be respectful and professional. Unprofessional behavior, including abusive or discriminatory conduct, undermines public trust and must be avoided. In addition, students should know how to manage inappropriate contact professionally. This content has been emphasized in both the traditional professionalism paradigm and in guidelines on professional use of SM by various governing bodies (48, 61). In this domain, the item “I redirect patients seeking medical advice on SM to appropriate in-person care” was excluded. While this statement aligns with international PSM standards, its exclusion may mask important social and cultural nuances in the Vietnamese context. In Vietnam, it is usually socially acceptable for health professionals to provide informal advice via SM platforms like Facebook or Zalo. This reflects a communal culture in which helping other people within a social network is highly valued (62). As a result, health professionals may feel a moral obligation to respond when someone approaches them for health-related concerns, and redirecting to formal care might be perceived as impolite, uncaring, or dismissive.

The domain “Practitioner’s privacy” consisted of competencies related to protecting physician identity and managing the visibility of personal information on SM. These competencies reflect the growing recognition that safeguarding a healthcare physician’s online presence in SM is essential to preserving professionalism and minimizing risks in digital spaces (21). This content has been included in multiple global guidelines. The AMA and World Medical Association, for instance, emphasize that physicians should utilize privacy settings to safeguard personal information on SM platforms (41, 48). Collectively, these insights affirm that safeguarding a physician’s online presence is not merely about personal privacy but is also integral for preserving the professionalism and integrity of the medical field in SM. However, during the Delphi process, the item “I understand that content I share online can be permanent even if I delete it from my account” was excluded from the final consensus. Panelists acknowledged the item’s relevance in principle but did not deem it a priority competency for Vietnamese medical students at this stage.

The “Health advocacy” domain underscores the significant role that medical students play in promoting public health and well-being through SM platforms. SM can be a powerful tool for healthcare professionals to engage with the public, but it also comes with the responsibility to act ethically (41). This is consistent with the expanding understanding of how health professionals can properly use SM to shape public health discourse (63). Notably, two items, “I understand that the use of SM may cause the conflict of interest” and “I manage the sites based on understanding about conflict of interest caused by SM use,” were excluded; this exclusion may reflect limited awareness or perceived irrelevance of conflicts of interest among panelists. Moreover, panelists do not consider these competencies as important, owing to the assumption that medical students are not influencers or public figures whose endorsements carry professional weight.

The domain “Information appropriateness” emphasizes that it is important for medical students to ensure that any information they share online is accurate, evidence-based, and contextually appropriate. Healthcare professionals are expected to provide accurate, scientifically supported medical information to the public. The dissemination of unverified or misleading information can have harmful consequences, especially in areas like public health, disease prevention, and treatment options. The World Health Organization emphasizes that healthcare professionals must rely on credible, evidence-based sources when communicating medical information to the public to prevent the spread of misinformation (64). This can be applied not only in face-to-face interaction but also in the online context of SM. During the Delphi process, the item “I actively monitor medical information on SM” was excluded from the list; this exclusion may reflect concerns about feasibility. Panelists may view this task to be beyond the expected role or capacity of medical students, especially in Vietnam, where students face heavy academic loads and may lack the authority or confidence to correct misinformation publicly. Furthermore, hierarchical norms within the Vietnamese healthcare system may discourage students from publicly correcting or critiquing information shared by senior practitioners or popular health influencers.

Compared with previous studies, our findings provide a specific, unique list of competencies focused on PSM, tailored to the Vietnamese context. Earlier studies have identified SM competencies or outlined principles for sharing health-related information online (31, 65). However, these were either general competencies related to SM use or addressed only limited issues, such as patient information disclosure, while overlooking many other aspects of professionalism. In contrast, our study offers a more comprehensive framework that encompasses all dimensions of PSM, ranging from patient confidentiality, professional boundaries, and information appropriateness to health advocacy on SM and practitioners’ privacy.

Our findings identify core PSM competencies to develop training programs on professionalism in the online environment at Vietnamese medical schools. These can enhance existing programs on traditional medical professionalism and provide an education framework to focus on addressing unique aspects of professionalism when using SM. Since all the competencies in the final list are deemed essential, medical schools and institutions could initially develop a curriculum focusing on the competencies with the highest consensus until all have been addressed (66). These competencies can be integrated into existing courses or incorporated as learning outcomes for seminars, workshops, or classes to enhance students’ awareness of the professional use of SM. Furthermore, reflective practice and scenario-based role-play could help address the challenges posed by SM to young medical students and strengthen self-regulation. The organization should have a team dedicated to developing and enhancing the quality of these programs to keep updated with the rapid growth of SM and digital media in general. The items excluded in the first Delphi round may be considered less significant in the current Vietnamese medical context. Nevertheless, unprofessional issues related to these items may still arise, suggesting that they should be incorporated into educational programs alongside key professional competencies. Moreover, as SM continues to evolve and expand, the competencies identified through the Delphi process should be periodically reviewed and updated.

This Delphi study has several limitations. First, it used purposive sampling rather than random sampling because it relies on participants’ expertise and work positions. All panelists were from one medical school (Hue UMP) which may limit wider regional representation and introduce potential institutional bias. Although the number of participants was within the acceptable range for the Delphi technique, increasing it to include participants from other medical institutions and organizations could further diversify the findings. Therefore, these findings are applicable only to this particular institute. Future studies could include a more diverse expert panel drawn from multiple institutions, as well as medical students or patients as panelists, to broaden perspectives and enhance generalizability. However, owing to the rigorous study design and comprehensive review process, the authors believe that the identified competencies have relevance for broader application in basic medical education regarding PSM beyond Hue UMP. Second, while the analysis accounted for adequate levels of CVR, degree of convergence, and degree of consensus, the additional comments from panelists were also incorporated, which may have introduced unintentional subjectivity. As such, future research must further validate and refine the proposed competency framework. Finally, PSM competencies identified in this Delphi study were confined to the undergraduate medical education level, highlighting the need to develop corresponding frameworks for postgraduate training.

In conclusion, this study defines a validated framework of SM professionalism competencies for Vietnamese medical students. The competency list identified in this Delphi survey can be a valuable contribution to the emerging field of professionalism in medical education and can serve as a reference for medical educators and policymakers when developing such a framework or educational interventions. While context-specific to Vietnam, the domains are aligned with universal professionalism principles and may inform curricula in similar educational settings. Future studies could broaden the application of these competencies by outlining detailed guidance on how to achieve them, including appropriate educational content, teaching methods, and assessment strategies.

## Data Availability

The raw data supporting the conclusions of this article will be made available by the authors, without undue reservation.
